# Parasitoid–host interaction behaviors in relation to host stages in the *Tamarixia triozae* (Hymenoptera: Eulophidae)*–Bactericera cockerelli* (Hemiptera: Triozidae) system

**DOI:** 10.1093/jisesa/ieae016

**Published:** 2024-02-25

**Authors:** Chen Chen, Xiong Z He, Peng Zhou, Qiao Wang

**Affiliations:** School of Life Sciences, Anhui Key Laboratory of Biodiversity Research and Ecological Protection in Southwest Anhui Province, Anqing Normal University, Anqing 246133, China; School of Agriculture and Environment, Massey University, Palmerston North 11222, New Zealand; School of Life Sciences, Anhui Key Laboratory of Biodiversity Research and Ecological Protection in Southwest Anhui Province, Anqing Normal University, Anqing 246133, China; School of Agriculture and Environment, Massey University, Palmerston North 11222, New Zealand

**Keywords:** behavior, handling time, host feeding, oviposition, host defense

## Abstract

Females of host-feeding parasitic wasps often partition hosts of different stages for feeding and parasitization, but the underlying behavioral mechanisms are largely unknown, making it difficult to evaluate parasitoid–host interactions and their effects on biological control success. *Tamarixia triozae* (Burks) is an ectoparasitoid of tomato-potato psyllid *Bactericera cockerelli* (Šulc), which utilizes nymphs and kills them both by parasitization and host feeding. In this study, we exposed female wasps to 1st- to 5th-instar psyllid nymphs simultaneously and made 13-h continuous video recording of parasitoid–host interactions. We then quantified host stage-dependent handling time for feeding and oviposition and behaviors of parasitoid attacks and host defenses from encountering to successful feeding and oviposition. Female wasps were more likely to encounter and evaluate older hosts. However, the encounter and evaluation did not necessarily result in the success of feeding and oviposition. Our findings suggest that (i) *T. triozae* continues to assess the host using its ovipositor after the evaluation phase, (ii) females prefer the mid-aged hosts for feeding piercing and feeding and the later instars for oviposition probing and oviposition, (iii) the combination of stage-specific host nutrition value, integument thickness and defense behavior determines the success of feeding attacks, and (iv) the optimal host resource for parasitoid offspring fitness defines host stage selection for oviposition. This study contributes to our understanding of parasitoid–host interactions and mechanisms behind host stage selections.

## Introduction

The optimal foraging theory predicts that parasitoids should adopt the best possible host selection strategies to maximize their lifetime fitness gain (Goubault et al. 2003, Danchin et al. 2008). In the field, parasitoid females often encounter their hosts of various stages, each with different nutrition value and defense ability (Gross 1993, Cadée and van Alphen 1997, Colinet et al. 2005, He et al. 2011, Khatri et al. 2016). Consequently, female wasps are predicted to behave differently when facing hosts of different stages. In host-feeding parasitoids, mothers need to balance their host selection for the fitness of both their offspring and their own (Heimpel and Collier 1996, Lauzière et al. 2001, Hanan et al. 2015). However, behavioral mechanisms behind the host stage selection for feeding and oviposition are unknown for most parasitoid–host systems, making it difficult to evaluate parasitoid–host interactions in relation to host stages and their effects on biological control success.

The tomato-potato psyllid (TPP), *Bactericera cockerelli* (Šulc) (Hemiptera: Triozidae), is serious pest of cultivated solanaceous crops in the United States, Mexico, and New Zealand, causing substantial economic losses (Cranshaw 1994, Teulon et al. 2009, Crosslin et al. 2010, Butler and Trumble 2012, Rojas et al. 2015). It has also been established in Australia, Canada, and Ecuador, and its economic impact there is under evaluation (FAO 2017, WADPIRD 2018, Castillo Carrillo et al. 2019, Olaniyan et al. 2020). *Tamarixia triozae* (Burks) (Hymenoptera: Eulophidae) is an important ectoparasitoid of TPP, which kills TPP nymphs by both parasitization and host feeding (Martinez et al. 2015, Rojas et al. 2015). It is a diurnally active wasp (Chen et al. 2020), laying an egg on the ventral side of the host and piercing the soft integument area of the host with its ovipositor and feeding on the hemolymph exuding from the wound (Martinez et al. 2015). When all nymph instars are present simultaneously, older hosts are more likely to be parasitized and mid-aged or younger ones fed on (Chen et al. 2023), but the underlying behavioral mechanisms of these patterns are not clear.

Several authors have observed feeding and oviposition behaviors of *T. triozae* on TPP. For example, Vega-Chávez (2010) has given brief qualitative descriptions of host feeding actions and host responses. In a nonchoice setting, Caudillo Ruiz (2010) has provided a brief note of oviposition behavior on the 4th-instar nymphs and nymph responses to attacks. Also, on the 4th-instar nymphs, Martinez et al. (2015) have made detailed qualitative descriptions of feeding and oviposition behavior and calculated handling time for feeding and oviposition. However, none of these authors has quantified host stage-dependent encounter rate, handling time, and behavioral sequence and frequency of parasitoid–host interactions from encounter to successful feeding and oviposition, knowledge of which provides insight into behavioral mechanisms of host stage selections for feeding and parasitization. This information is important for the evaluation of the biological control effectiveness of this parasitoid under various seasonal host stage demographies.

The present study aimed to determine the host feeding and oviposition behaviors of *T. triozae* in relation to the host stage and defense behavior of TPP nymphs. We provided mated *T. triozae* females with TPP nymphs of all instars (1st–5th instars) at the same space and time and made a 13-h continuous video recording of parasitoid–host interaction behaviors during the photophase. We then quantified the handling time for feeding and oviposition on hosts of different stages and the host stage-dependent behaviors of parasitoid attacks and host defenses from encountering to successful feeding and oviposition. This study provides knowledge of behavioral mechanisms behind the host stage selection for future investigations into parasitoid–host interactions.

## Materials and Methods

### Breeding Colonies and Environmental Conditions

The colonies of TPP and *T. triozae* started from psyllid and wasp adults from BioForce Limited, Auckland, New Zealand. We maintained these 2 colonies in 2 separate climate rooms to ensure parasitoids did not contaminate the psyllid colony. We reared psyllids on 5 potted 2-month-old bell pepper plants (*Capsicum annuum* L.) in an aluminum-framed cage (43 × 42 × 40 cm) with a metal mesh (aperture size = 0.25 × 0.25 mm) on the back and both sides and Perspex (a solid transparent plastic made of polymethyl methacrylate) on the top and front, and aluminum alloy on the bottom. We reared wasps on the 3rd- to 5th-instar psyllid nymphs feeding on 5 potted 2-month-old bell pepper plants in another cage of the same size. We maintained the colonies and carried out experiments at 25 ± 1 °C and RH 40%–60% with a photoperiod of 14:10 h (L:D).

### Insects for Experiments

To obtain psyllid nymphs of all instars for this experiment, we randomly transferred 70 adults from the colony onto 1 potted 2-month-old bell pepper plant in an aluminum-framed cage described above and allowed them to stay in this cage. We replaced the plant infested with psyllid eggs with an un-infested one of the same age once every 24 h for 24 days to obtain sufficient psyllid nymphs of desirable instars for experiments. We individually maintained infested plants in a nylon-framed cage (65 × 50 × 50 cm, aperture size = 0.075 × 0.075 mm). When nymphs developed to the 5th instar on the 1st infested plant, we harvested all nymphs on the infested plants using a paintbrush to obtain insects of all instars at the same time. We identified and separated nymph instars under a stereomicroscope (Leica MZ12, Germany), according to Vega-Chávez (2010). We then transferred harvested nymphs into Petri dishes according to experimental design.

To obtain parasitoids for this experiment, we introduced 10 female parasitoids randomly collected from the colony into an above-mentioned nylon-framed cage, maintaining one 2-month-old bell pepper plant infested with 200 4th-instar psyllid nymphs. After 24 h, we removed all parasitoids using an aspirator. We collected parasitoid pupae 7 days later and individually placed them in cotton-plugged glass vials (5 cm in height × 1.5 cm in diameter) until adult emergence. We obtained 60 newly emerged adults (30 females and 30 males), allowed them to stay in the vials individually, and provided them with 10% honey solution saturated in cotton wool balls (0.5 cm in diameter) as food. Because both sexes become sexually mature within 24 h after emergence (Chen et al. 2020), we individually paired 1-day-old virgin males and females in the vials until mating ended. Mating usually occurs within 90 min after pairing and lasts for about 15 min (CC pers. observ.). We considered mating successful when paired females and males produced at least one daughter.

Because most female wasps start laying eggs when they are 3 days old (Rojas et al. 2015, Chen et al. 2022), we used 4-day-old mated females for experiments. We obtained 14 mated females for experiments. We treated each female as a replicate (a total of 14). Using an aspirator, we transferred each mated female into a Petri dish (8.5 cm in diameter × 2.4 cm in height) containing 30 host nymphs (6 individuals per instar × 5 instars) on a bell pepper leaf. We wrapped the leaf petiole with water-saturated cotton wool with parafilm to keep it fresh. The lid of the Petri dish had 2 holes (1 cm in diameter), one plugged with a cotton wool ball for transferring wasps and another covered with metal mesh (aperture size = 0.25 × 0.25 mm) for ventilation. We allowed the female to stay in a Petri dish for 24 h and then transferred it to another Petri dish with hosts as described above and allowed it to stay for 24 h. We repeated this process until the parasitoid was 4 days old before experiments. We provided water saturated in a cotton ball placed on the bottom of the Petri dish for the wasp and replaced it every day.

### Behavioral Recording

To determine the host feeding and oviposition behavior of the parasitoids in relation to the host stage and defense behavior of psyllid nymphs, we cut a bell pepper leaf into a square (5 cm × 2.5 cm) and placed the leaf square upside down on a 1%-agar block (5 cm × 2.5 cm × 0.5 cm) in the center of a Petri dish. We transferred 30 host nymphs (6 individuals per instar × 5 instars) using a paintbrush onto the bell pepper leaf square 30 min after lights-on. We randomly distributed the nymphs of different instars on the leaf square. Forty minutes after lights-on, we introduced a 4-day-old mated and experienced female parasitoid prepared above into the Petri dish and continuously recorded behaviors of both the parasitoid and its hosts of different instars in each dish for 13 h (40 min after lights-on to 20 min before lights-off) using a digital video camera (Sony Handycam HDR-CX405, Japan). We repeated the recordings in 14 Petri dishes (14 parasitoids and 420 nymphs). After behavioral recording for each dish, we examined all nymphs for evidence of feeding (Morales et al. 2013, Martinez et al. 2015) and oviposition under a stereomicroscope (Chen et al. 2023). We watched the video recorded for each dish, inputted each behavioral event into the dataset, and calculated the mean values of each behavioral event for each dish.

For each female of the parasitoid, we recorded encounter, evaluation, piercing for feeding, feeding, handling time for feeding, oviposition probing, oviposition, and handling time for oviposition. For psyllid defense behavior, we recorded swaying and escaping. Detailed descriptions of psyllid behavior can be found in Table 1. We noted down the instar of the host that the wasp interacted with during the recording of these behaviors.

**Table 1.** Definition of host feeding and oviposition behaviors of *Tamarixia triozae* and defense behavior of *Bactericera cockerelli*

Behavior	Definition
**Parasitoid**	
Encounter	The number of times a parasitoid physically contacted a host
Evaluation	The number of times a parasitoid examined a host by walking on the host and touching it with its antennae before proceeding with the oviposition or feeding
Piercing for feeding	The number of times a parasitoid pierced the dorsal soft integument area of a host with its ovipositor
Feeding	The number of hosts of different instars fed by the parasitoid
Handling time for feeding	The period a parasitoid spent from the start of encountering a host to the completion of feeding
Oviposition probing	The number of times a parasitoid probed a host with its ovipositor between the host body and leaflet surface
Oviposition	The number of hosts parasitized
Handling time for oviposition	The period from the start of encountering to the completion of oviposition
**Host**	
Swaying	The number of times a host swayed its abdomen side to side to prevent attack by a parasitoid
Escaping	The number of times a host walked away when attacked by a parasitoid

### Statistical Analysis

We conducted all data analyses using SAS software (SAS 9.3, SAS Institute Inc., NC, USA) with a rejection level set at *P* = 0.05. We analyzed data on the number of encounters, handling time for feeding using a linear mixed model (PROC MIXED). We used a generalized linear model (PROC GLIMMIX) with a *log* function and Gamma distribution to analyze oviposition handling time and a Poisson distribution to analyze number of evaluations, feeding probing, host feeding, oviposition probing, oviposition, and number of escaping and swaying of host nymphs. The instar stage was a fixed factor, and the replicate was a random factor in these 2 models. We performed multiple comparisons between instar stages using the Tukey’s test.

## Results

### Description of Behaviors During Parasitoid–Host Interactions

**Fig. 1.**
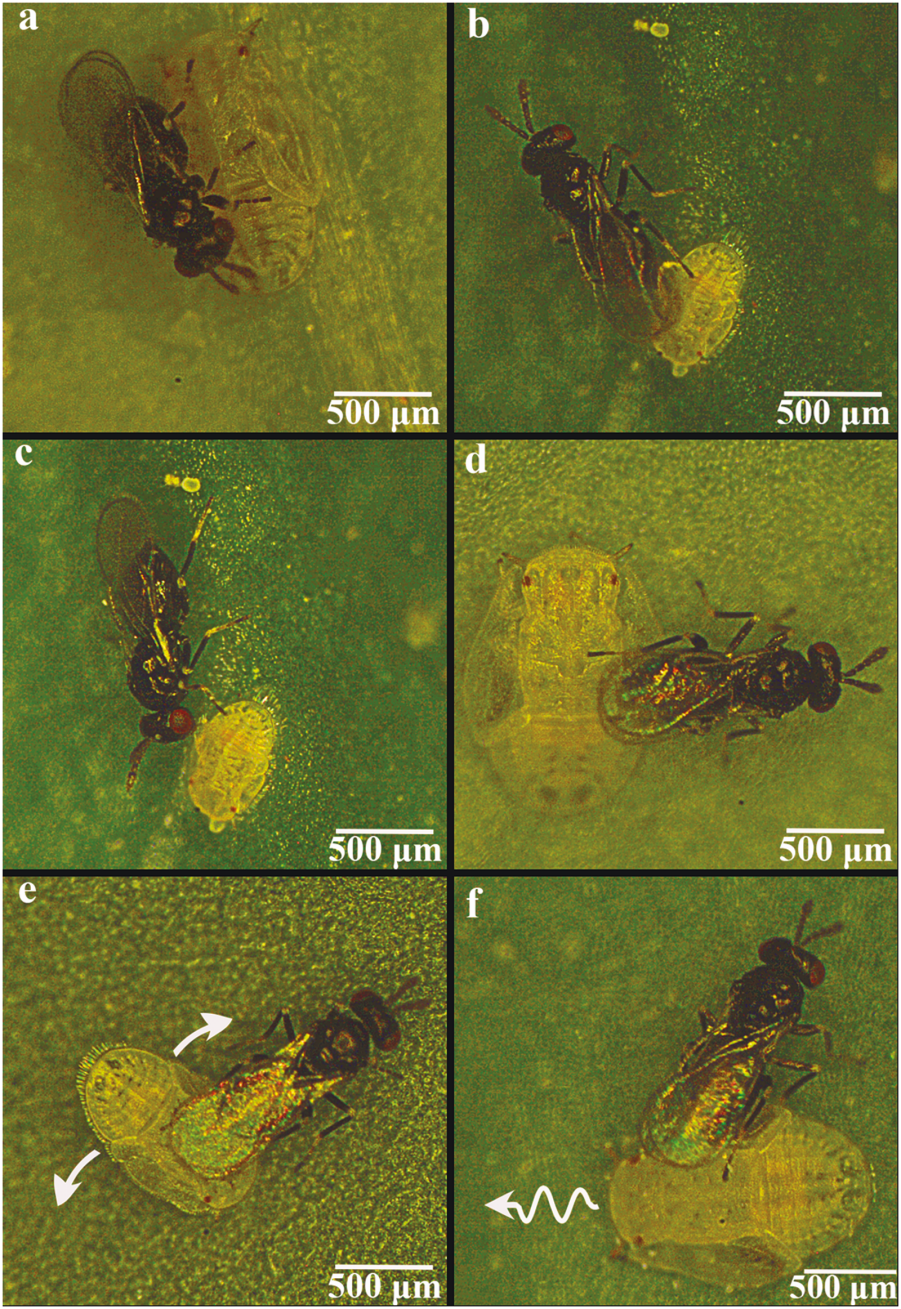
A–D) *Tamarixia triozae* attacking and E) and F) *Bactericera cockerelli* defense behaviors: A) evaluation, B) piercing for feeding, C) feeding, D) oviposition probing, E) swaying to avoid attack, and F) escaping to avoid attack. The arrows in E) and F) indicate the direction of movement.

On average, the parasitoids started foraging on the bell pepper leaf square after about 1 h (64.01 ± 18.09 min, mean ± SE) of being placed in the Petri dish. Upon physical contact with a host, the female evaluated the host by walking on and frequently touching it with antennae (Fig. 1A). Once a host was selected for feeding, the parasitoid pierced the soft integument area on the dorsal side of the host body with its ovipositor to make a wound (Fig. 1B) and then fed on the extravasated hemolymph (Fig. 1C). When the parasitoid chose to parasitize a host, it probed the host with its ovipositor between the host body and leaf surface and then laid egg(s) (Fig. 1D). Eggs were usually deposited on the ventral side of the host between the prothoracic and mesothoracic legs or the mesothoracic and metathoracic legs. Host defense behavior to avoid attack included swaying its abdomen side to side (Fig. 1E) and walking away (Fig. 1F). Videos of host defense behavior are provided as Supplementary Material.

### Host Stage-Dependent Encountering, Evaluation, Feeding, and Oviposition

**Fig. 2.**
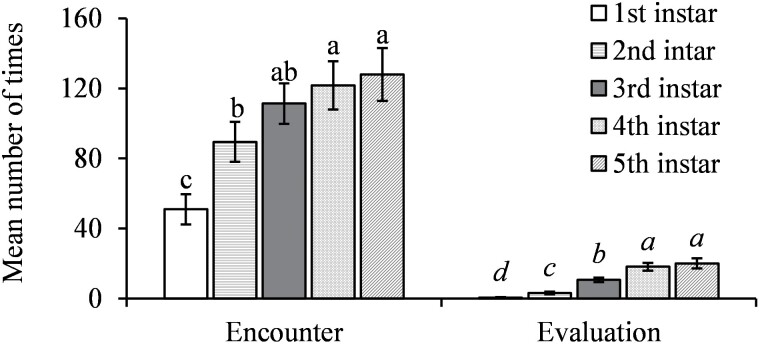
Mean (±SE) number of encounters and evaluations by *Tamarixia triozae* on *Bactericera cockerelli* nymphs of different instars. For each category on the *x*-axis (distinguished by italic and nonitalic letters), columns with different letters are significantly different (*α* = 0.05).

**Fig. 3.**
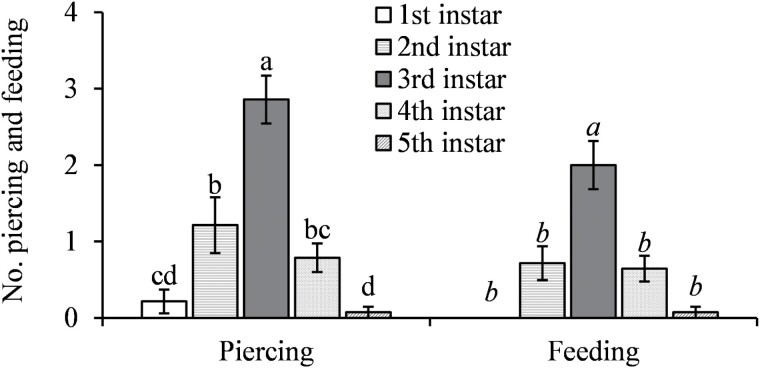
Mean (±SE) number of piercings for feeding and hosts fed by *Tamarixia triozae* on *Bactericera cockerelli* nymphs of different instars. For each category on the *x*-axis (distinguished by italic and nonitalic letters), columns with different letters are significantly different (*α* = 0.05).

**Fig. 4.**
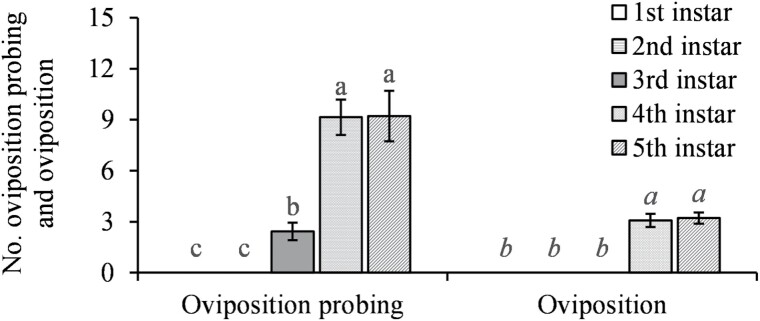
Mean (±SE) number of oviposition probings and hosts parasitized by *Tamarixia triozae* on *Bactericera cockerelli* nymphs of different instars. For each category on the *x*-axis (distinguished by italic and nonitalic letters), columns with different letters are significantly different (*α* = 0.05).

The parasitoids were significantly more likely to encounter and evaluate the older hosts (*F* = 14.84; *df* = 4, 52; *P* < 0.0001 for encounter; *F* = 59.69; *df* = 4, 52; *P* < 0.0001 for evaluation) (Fig. 2). They performed feeding piercing on all instars they encountered but were significantly more likely to pierce and feed on the 3rd-instar nymphs (*F* = 10.52; *df* = 4, 52; *P* < 0.0001 for piercing; *F* = 5.01; *df* = 4, 52; *P* = 0.0017 for host feeding) with no feeding on the 1st-instar nymphs (Fig. 3). The parasitoids performed oviposition probing on the 3rd- to 5th-instar nymphs with a preference of 4th and 5th over 3rd instars for oviposition probing and only laid eggs under 4th and 5th instars (*F* = 42.38; *df* = 4, 52; *P* < 0.0001 for oviposition probing; *F* = 15.59; *df* = 4, 52; *P* < 0.0001 for parasitism) (Fig. 4).

### Host Stage-Dependent Defense Response and Handling Time for Feeding and Oviposition

**Fig. 5.**
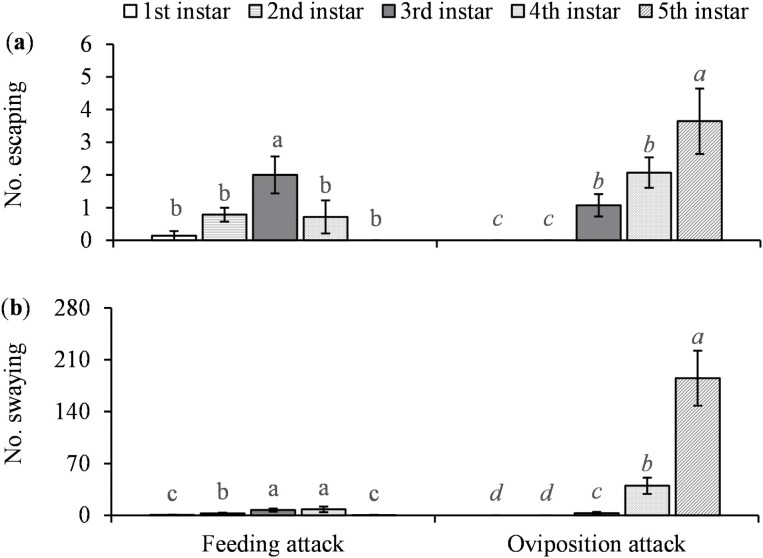
Defense behavior of *Bactericera cockerelli* nymphs of different instars in response to feeding and oviposition attacks by *Tamarixia triozae*: A) mean (±SE) number of escapes and B) mean (±SE) number of sways. For each category on the *x*-axis (distinguished by italic and nonitalic letters), columns with different letters are significantly different (*α* = 0.05).

**Fig. 6.**
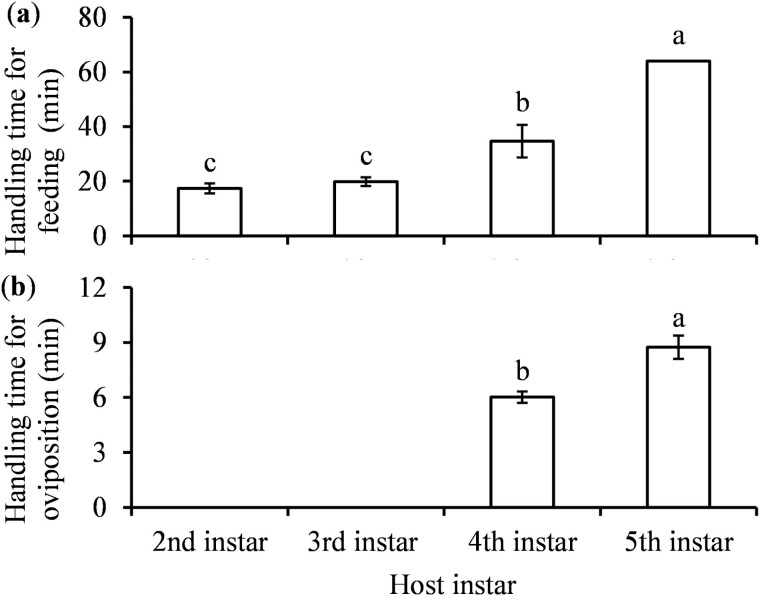
Mean (±SE) handling time for A) feeding and B) oviposition by *Tamarixia triozae* on *Bactericera cockerelli* nymphs of different instars. Columns with different letters are significantly different (*α* = 0.05).

In response to parasitoid attack for feeding, the 3rd-instar nymphs escaped attack by parasitoids significantly more frequently than other host stages (*F* = 4.75; *df* = 4, 52; *P* = 0.0024) (Fig. 5A), and the 3rd- and 4th-instar nymphs swayed significantly more frequently than other host stages (*F* = 30.54; *df* = 4, 52; *P* < 0.0001) (Fig. 5B). The 5th-instar nymphs were significantly more likely to defend themselves from oviposition attack by escaping and swaying than the 3rd and 4th instars (*F* = 12.33; *df* = 4, 52; *P* < 0.0001 for escape times; *F* = 606.66; *df* = 4, 52; *P* < 0.0001 for sway times) (Fig. 5). Wasps spent significantly longer time to handle the 4th- and 5th-instar nymphs than the 2nd- and 3rd-instar nymphs for feeding (*F* = 10.88; *df* = 3, 32; *P* < 0.0001) (Fig. 6A). They also spent significantly longer time to handle the 5th-instar nymphs than the 4th instars for oviposition (*F* = 19.73; *df* = 1, 83; *P* < 0.0001) (Fig. 6B).

## Discussion

Our results show that *T. triozae* females were more likely to encounter and evaluate older hosts but preferred mid-aged hosts for feeding piercing and feeding and late instars for oviposition probing and oviposition. Late instar hosts were more likely to avoid oviposition attacks by escaping and swaying. The higher probability of encountering and evaluating late instar hosts (4th and 5th instars) could be attributed to the fact that parasitoids can use visual and/or chemosensory cues to locate hosts in a short-range (Battaglia et al. 1995, 2000, Mackauer et al. 1996, Powell et al. 1998, Weinbrenner and Volkl 2002, He et al. 2011) and older hosts are more visible and emit more semiochemicals (Hanan et al. 2015, Khatri et al. 2016, Bui et al. 2020). The behavioral divergence occurred after the evaluation phase: the females preferred to pierce and feed on mid-aged nymphs (3rd instar) and probe and parasitize late instar ones (4th and 5th instars). Similar to a congeneric species *Tamarixia radiata* (Waterston) (Hymenoptera: Eulophidae) (Chen and Stansly 2014), host feeding and oviposition in *T. triozae* is nonconcurrent, i.e., females do not feed on parasitized hosts or parasitize fed ones (Cerón-González et al. 2014, Chen et al. 2023), our findings suggest that this parasitoid has developed a host partitioning strategy for feeding and parasitization to minimize the competition for hosts between mothers and their offspring, in accordance with the optimal foraging theory (Kishani Farahani et al. 2015) which predicts that parasitoids should adopt the best possible host selection strategies to maximize their lifetime fitness gain (Goubault et al. 2003, Danchin et al. 2008). This strategy may allow *T. triozae* to control TPP effectively by killing hosts of different life stages simultaneously.

We showed that piercing the 1st-instar nymphs and probing under the 3rd-instar ones did not result in successful feeding and oviposition, respectively. These findings suggest that *T. triozae* continues to assess the host using its ovipositor during piercing or probing because some, specifically eulophid parasitoids, carry the sensilla on their ovipositors that may play key roles in host stage recognition (Huang et al. 2019, Wong et al. 2021). The reduced feeding piercing and feeding on younger and late instar nymphs may result from the cost–benefit assessment by the wasp. Younger hosts have thinner integuments, which are easier to penetrate (Kidd and Jervis 1991, Veronesi et al. 2022) but have less nutrition value (Kidd and Jervis 1991, Fellowes et al. 2007, Sule et al. 2014, Hanan et al. 2015). Although *T. triozae* occasionally parasitizes the 3rd-instar nymphs, no female offspring emerge from hosts of this instar (Chen et al. 2023), suggesting that hosts younger than the 3rd instar do not have sufficient resources to sustain the parasitoid population because daughter production is necessary to sustain the population.

Although the 3rd-instar nymphs performed significantly more swaying and escaping in response to feeding attacks, the wasps were significantly more likely to pierce and feed on the hosts of this stage. On the other hand, the parasitoids spent significantly more time handling the 5th-instar hosts but had significantly less success in piercing and feeding. These findings imply that the combination of age-specific host defense behavior and integument thickness might determine the ultimate outcome of feeding attacks, and this parasitoid selects to feed on mid-aged hosts to maximize their own fitness. In response to the oviposition attack, host defense activities and parasitoid handling time significantly increased with the increase of host stages. However, the parasitoids were still significantly more likely to perform oviposition probing on and parasitize the 4th- and 5th-instar hosts. The results suggest that *T. triozae* selects to parasitize older hosts regardless of host defense to maximize the fitness of their offspring, as reported in Chen et al. (2023).

Overall, *T. triozae* females were more likely to encounter and evaluate older hosts under the conditions of these experiments due to their larger size and probably also stronger chemical cues. However, the encounter and evaluation frequencies did not necessarily result in feeding and oviposition success. They preferred the 3rd instar for feeding and the 4th and 5th instars for oviposition regardless of stage-specific host defense behavior. Such host partitioning strategies may maximize the fitness of both *T. triozae* adults and their offspring. We suggest that the combination of age-specific host nutrition value, integument thickness, and defense behavior governs the success of feeding attacks while the optimal resource for offspring fitness explains host stage selection for oviposition. Our findings could be useful for further understanding parasitoid–host interactions and for the evaluation of biological control effectiveness under various seasonal host stage demographies.

## Supplementary Material

ieae016_suppl_Supplementary_Material

## Data Availability

The datasets from the current study are available from the corresponding author on request.
